# Late functional improvement after lacunar stroke: a population-based study

**DOI:** 10.1136/jnnp-2018-318434

**Published:** 2018-07-21

**Authors:** Aravind Ganesh, Sergei A Gutnikov, Peter Malcolm Rothwell, for the Oxford Vascular Study, Rose M Wharton

**Affiliations:** Centre for Prevention of Stroke and Dementia, Nuffield Department of Clinical Neurosciences, University of Oxford, Oxford, UK

**Keywords:** ischaemic stroke, functional outcome, lacunar strokes, recovery, rehabilitation, trail design

## Abstract

**Background:**

Recovery in function after stroke involves neuroplasticity and adaptation to impairments. Few studies have examined differences in late functional improvement beyond 3 months among stroke subtypes, although interventions for late restorative therapies are often studied in lacunar stroke. Therefore, we compared rates of functional improvement beyond 3 months in patients with lacunar versus non-lacunar strokes.

**Methods:**

In a prospective, population-based cohort of 3-month ischaemic stroke survivors (Oxford Vascular Study; 2002–2014), we examined changes in functional status (modified Rankin Scale (mRS), Rivermead Mobility Index (RMI), Barthel Index (BI)) in patients with lacunar versus non-lacunar strokes from 3 to 60 months poststroke, stratifying by age. We used logistic regression adjusted for age, sex and baseline disability to compare functional improvement (≥1 mRS grades, ≥1 RMI points and/or ≥2 BI points), particularly from 3 to 12 months.

**Results:**

Among 1425 3-month survivors, 234 patients with lacunar stroke did not differ from others in 3-month outcome (adjusted OR (aOR) for 3-month mRS >2 adjusted for age/sex/National Institutes of Health Stroke Scale score/prestroke disability: 1.14, 95% CI 0.75 to 1.74, p=0.55), but were more likely to demonstrate further improvement between 3 months and 1 year (aOR (mRS) adjusted for age/sex/3-month mRS: 1.64, 1.17 to 2.31, p=0.004). The results were similar on restricting analyses to patients with 3-month mRS 2–4 and excluding recurrent events (aOR (mRS): 2.28, 1.34 to 3.86, p=0.002), or examining BI and RMI (aOR (RMI) adjusted for age/sex/3-month RMI: 1.78, 1.20 to 2.64, p=0.004).

**Conclusion:**

Patients with lacunar strokes have significant potential for late functional improvement from 3 to 12 months, which should motivate patients and clinicians to maximise late improvements in routine practice. However, since late recovery is common, intervention studies enrolling patients with lacunar strokes should be randomised and controlled.

## Introduction

Functional improvement after neurological lesions like stroke or demyelination is driven by neural recovery, through structural and functional plasticity,^1^ and/or by the individual’s physiological and psychosocial adaptation to activities with residual impairments.^2 3^ Although the capacity for neuroplasticity is known to be influenced by the nature of the initial injury,^4^ the differential implications of lesion location and stroke subtype for overall functional improvement remain uncertain. For instance, one might consider patients with lacunar strokes, affecting subcortical structures,^5^ as potentially having a greater capacity for functional improvement than those with non-lacunar strokes, given their intact cortex recruitable for plasticity and adaptive strategies. On the other hand, the damage to densely packed tracts might leave patients with lacunar stroke less capable of meaningful improvement despite cortical plasticity or adaptation. Some studies suggest that certain lacunar syndromes might have a greater capacity for recovery,^6^ while others report no differences in recovery at discharge in cortical or subcortical strokes.^7^ Of the few studies that have examined recovery beyond the acute phase, one found no differences,^8^ while another suggested that patients with lacunar stroke fare worse,^9^ but they were limited by small sample size and/or retrospective design. Most studies have also focused on neurological recovery (improvement in specific impairments like motor strength), but elucidating changes in functional improvement (daily activities) is also clinically relevant, as both recovery and adaptive capabilities could be harnessed in rehabilitation.

The potential for patients with lacunar strokes to demonstrate late improvement has also been suggested in some studies of restorative therapies that specifically enrolled patients with small subcortical strokes,^10–15^ although not all had a control group.^13–15^ However, given the paucity of published data on differences between stroke subtypes in late recovery trajectories, it is uncertain how much late improvement can be expected simply on the basis of untreated natural history.^16^ To better inform patients and clinicians about prognosis in routine practice, and inform the design and interpretation of rehabilitation studies, we compared functional improvement beyond 3 months in patients with lacunar versus non-lacunar strokes in a prospective, population-based cohort (Oxford Vascular Study, OXVASC).

## Methods

The OXVASC population comprises 92 728 patients registered with about 100 general practitioners (GPs) in 9 practices across Oxfordshire. The study methods have been published.^17^ Recruitment has been ongoing since April 2002. Patients with suspected transient ischaemic attack (TIA) or stroke are ascertained using overlapping methods of ‘hot’ and ‘cold’ pursuit, including daily rapid-access ‘TIA/stroke clinic’ to which participating GPs and the local emergency department (ED) refer all unhospitalised individuals with suspected TIA/stroke; daily searches of ward admissions (medical, cardiology, stroke, neurology), ED attendance register and in-hospital bereavement office death records; and monthly searches of death certificates, coroners’ reports (for out-of-hospital deaths), GP and hospital diagnostic/discharge codes, and brain/vascular imaging referrals. Direct assessment has shown ascertainment is near complete.^18^

Patients with ischaemic stroke recruited from April 2002 to March 2014 were included. Patients were assessed urgently by study clinicians and considered for inclusion. Stroke was diagnosed per the WHO definition.^19^ Neurological impairment, medical history and risk factors were assessed. Stroke severity was measured using the National Institutes of Health Stroke Scale (NIHSS). All cases were reviewed by a senior neurologist (PMR) daily and imaging was reviewed by the study neuroradiologist. Patients received no interventions beyond standard care.

Patients had face-to-face follow-up with a study nurse/physician either in a hospital clinic or at home at 1 month, 3 months, 6 months, 1 year and 5 years. At each visit, functional status was assessed using the modified Rankin Scale (mRS), Rivermead Mobility Index (RMI) and Barthel Index (BI). The mRS is a 7-point disability scale ranging from 0 (no symptoms) to 6 (death).^20^ The RMI assesses 15 functional mobility tasks and ranges from 0 (cannot perform any) to 15 (can perform all).^21^ The BI assesses activities of daily living and ranges from 0 (dependent for all) to 20 (independent for all).^22^ These scales are often used as outcome measures in trials of poststroke restorative therapies.^23^ Raters were trained in mRS assessment using an instructional DVD with written materials produced by the University of Glasgow, previously used in large-scale trials,^20^ and underwent additional training and observation for RMI and BI assessments. Prestroke mRS and BI were determined at enrolment.

Patients who moved out of the study area were followed up by telephone. Additional information was obtained from carers in patients with significant speech or cognitive impairment. Recurrent vascular events were identified by daily OXVASC ascertainment, follow-up interviews and review of GP/hospital diagnostic codes. All deaths were also recorded from death certificates, coroners’ reports and the National Health Service Central Register. Poststroke healthcare resource use was obtained until 1 May 2017, including hospital-based rehabilitation (with length of stay, LOS) and community-based rehabilitation (physiotherapy, speech/language, occupational therapy).

### Statistical analyses

Only patients surviving ≥3 months after their first (‘index’) stroke in the study period were included to focus on functional improvement beyond the 90-day endpoint favoured by acute stroke trials. Analyses were censored at 1 May 2017.

We classified strokes as lacunar/non-lacunar using the TOAST (Trial of Org 10172 in Acute Stroke Treatment) criteria for small-vessel occlusion.^24^ As sensitivity analyses, we also examined recovery trends in patients with lacunar stroke syndrome (LACS) per the Oxfordshire Community Stroke Project classification who did not necessarily meet the TOAST criteria for small-vessel aetiology.^25^

We plotted 3-month mRS against the initial NIHSS score to examine early functional improvement in the first 3 months poststroke. We then examined functional improvement beyond 3 months by plotting changes in mRS from 3 months to 6 months, 6 months to 1 year, and from 1 year to 5 years for patients with lacunar versus non-lacunar strokes, further stratifying patients by age (<75 and >75 years).

Any drop in mRS is meaningful, as long-term mortality and dependency rise with each scale increment,^26^ so patients were deemed to show functional improvement if the score decreased by ≥1 grades. Logistic regression was used to model the association of lacunar versus non-lacunar stroke with mRS improvement in each time-period, adjusted for age/sex/baseline score for that time-period (eg, 3-month mRS for improvement from 3 months to 1 year; 1-year mRS for 1–5 years). Patients with mRS=0 at the beginning of each time-period were excluded from respective regressions since they could not show improvement. To minimise bias of the 1-year functional improvement analysis in favour of patients with lacunar strokes from their lower mortality, while avoiding survivorship bias, we used the most recent mRS prior to death (ie, 6-month mRS) whenever available for patients who died within 1 year, and otherwise excluded 1-year deaths. To focus on patients with mild-to-moderate disability who might be recruited in rehabilitation trials, we repeated the analysis using only patients with 3-month mRS 2–4. To verify that differences were not reflecting non-stroke-related disability, we repeated these regressions, progressively excluding patients with recurrent vascular events, prestroke mRS >2 and relevant comorbidities (peripheral vascular disease, heart failure, valve disease, cancer).

We validated our findings by repeating these analyses with the RMI and BI. The RMI’s minimal clinically important difference (MCID) is not established, but test–retest studies suggest that increases by ≥1 points are reliable, so this was deemed indicative of functional improvement on logistic regression.^27^ The BI’s MCID is 1.85 points, so increases by ≥2 points were deemed indicative of functional improvement.^22^ Patients with 3-month RMI=15 and BI=20 were excluded as they could not show improvement.

Statistical analyses used STATA V.13.1. Trends in ordinal data were compared using non-parametric Wilcoxon rank-sum tests corrected for ties, and dichotomous variables were compared using χ^2^ tests. Significance was set at p<0.050.

## Results

Of 1606 patients with ischaemic stroke, 181 (11.3%) died within 3 months. Baseline data were available for 1403 (98.5%) of the 1425 3-month survivors. Patients with lacunar stroke were younger than non-lacunar stroke patients, more often men, had lower initial NIHSS score and were less likely to have premorbid disability (table 1). There was no difference between patients with lacunar and non-lacunar strokes in thrombolysis (NIHSS adjusted OR (aOR): 0.67, 95% CI 0.08 to 5.44, p=0.71) or community-based rehabilitation (NIHSS aOR: 0.97, 0.63 to 1.49, p=0.88). Although patients with lacunar stroke seemed less likely to receive hospital-based rehabilitation (table 1), this difference was no longer seen on adjusting for stroke severity (NIHSS aOR: 0.77, 0.53 to 1.12, p=0.18; mean LOS if NIHSS score ≥5: lacunar=54.9 days, non-lacunar=63.9, p=0.57). Over 5 years of follow-up, there were fewer deaths among lacunar strokes but no differences in recurrent vascular events or poststroke depression (table 1; flow diagram in online supplementary figure I). Complete mRS data were available for 1403 (98.5%) and RMI/BI data for 1228 (86.2%) 3-month survivors. There was no difference between lacunar and non-lacunar stroke patients with missing RMI/BI data (n=197) in the distribution of NIHSS scores (p_trend_=0.58), 3-month mRS (p_trend_=0.14) or improvement on mRS from 3 months to 1 year (p_trend_=0.36).

10.1136/jnnp-2018-318434.supp1 Supplementary data

**Table 1:** Characteristics of 3-month survivors of ischaemic stroke

Characteristics	Lacunar stroke (n=234)	Non-lacunar stroke (n=1191)	P values
Age, mean (SD)	69.9 (12.0)	73.8 (12.8)	<0.001*
Sex (male) (%)	143 (61.1)	610 (51.2)	0.006*
History (%)			
Myocardial infarction	13 (5.6)	164 (13.8)	<0.001*
Angina	32 (13.7)	207 (17.4)	0.17
Atrial fibrillation	2 (0.9)	258 (21.7)	<0.001*
Hypertension	145 (62.0)	744 (62.5)	0.89
Dyslipidaemia	72 (30.8)	397 (33.3)	0.45
Diabetes	42 (17.9)	163 (13.7)	0.09
Peripheral vascular disease	13 (5.6)	95 (8.0)	0.20
Stroke	22 (9.4)	136 (11.4)	0.37
Transient ischaemic attack	29 (12.4)	176 (13.8)	0.34
Smoking	147 (62.8)	689 (57.9)	0.16
Heart failure	4 (1.7)	115 (9.7)	<0.001*
Valvular heart disease	14 (6.0)	120 (10.1)	0.05
Cancer	32 (13.7)	189 (15.9)	0.40
Prestroke mRS >2	17 (7.3)	227 (19.1)	<0.001*
Prestroke BI <20	38 (16.2)	280 (23.5)	0.06
InitialNIHSS score, mean (SD)	2.1 (2.2)	4.1 (5.4)	<0.001*
Received thrombolysis (%)	1 (0.5)	17 (1.6)	0.20
Received in-hospital rehabilitation (%)	43 (18.4)	385 (32.3)	<0.001*
Length of stay for rehabilitation, median days (IQR)	16 (5–43)	20 (5–69)	0.095
Received community-based rehabilitation (%)	28 (12.0)	160 (13.4)	0.54
Number of sessions, median (IQR)	2 (1–4.5)	2.5 (1–9)	0.28
Recurrent stroke within 5- years (%)	33 (14.1)	178 (15.0)	0.74
Any recurrent vascular event within 5 years (%)	64 (27.4)	296 (24.9)	0.42
Poststroke depression (%)	60 (25.6)	290 (24.4)	0.85
Deaths (%)			
Within 1 year	1 (0.4)	137 (11.5)	<0.001*
Within 5 years	41 (19.1)	423 (40.8)	<0.001*

*Significant differences at p<0.05. We compared ordinal/continuous variables using the Wilcoxon rank-sum (Mann-Whitney U) and dichotomous variables using χ^2^ tests.

Results in bold represent significant p values.

BI, Barthel Index; mRS, modified Rankin Scale; NIHSS, National Institutes of Health Stroke Scale.

Patients with lacunar stroke did not differ from non-lacunar strokes in 3-month functional improvement (OR for mRS >2 adjusted for age/sex/initial NIHSS score/thrombolysis/prior disability=1.14, 95% CI 0.75 to 1.74, p=0.55; online supplementary figures II, III). Three-month RMI and BI (online supplementary figures IV, V) were also not different after adjusting for age/sex/NIHSS score/prior disability (adjusted coefficient (RMI): 0.03, –0.48 to 0.53, p=0.92; adjusted coefficient (BI): 0.42, –0.18 to 1.02, p=0.17), and patients with lacunar stroke were no less likely to have 3-month RMI <15 or BI <20 (aOR: 1.14, 0.80 to 1.62, p=0.46).

**Figure 1:**
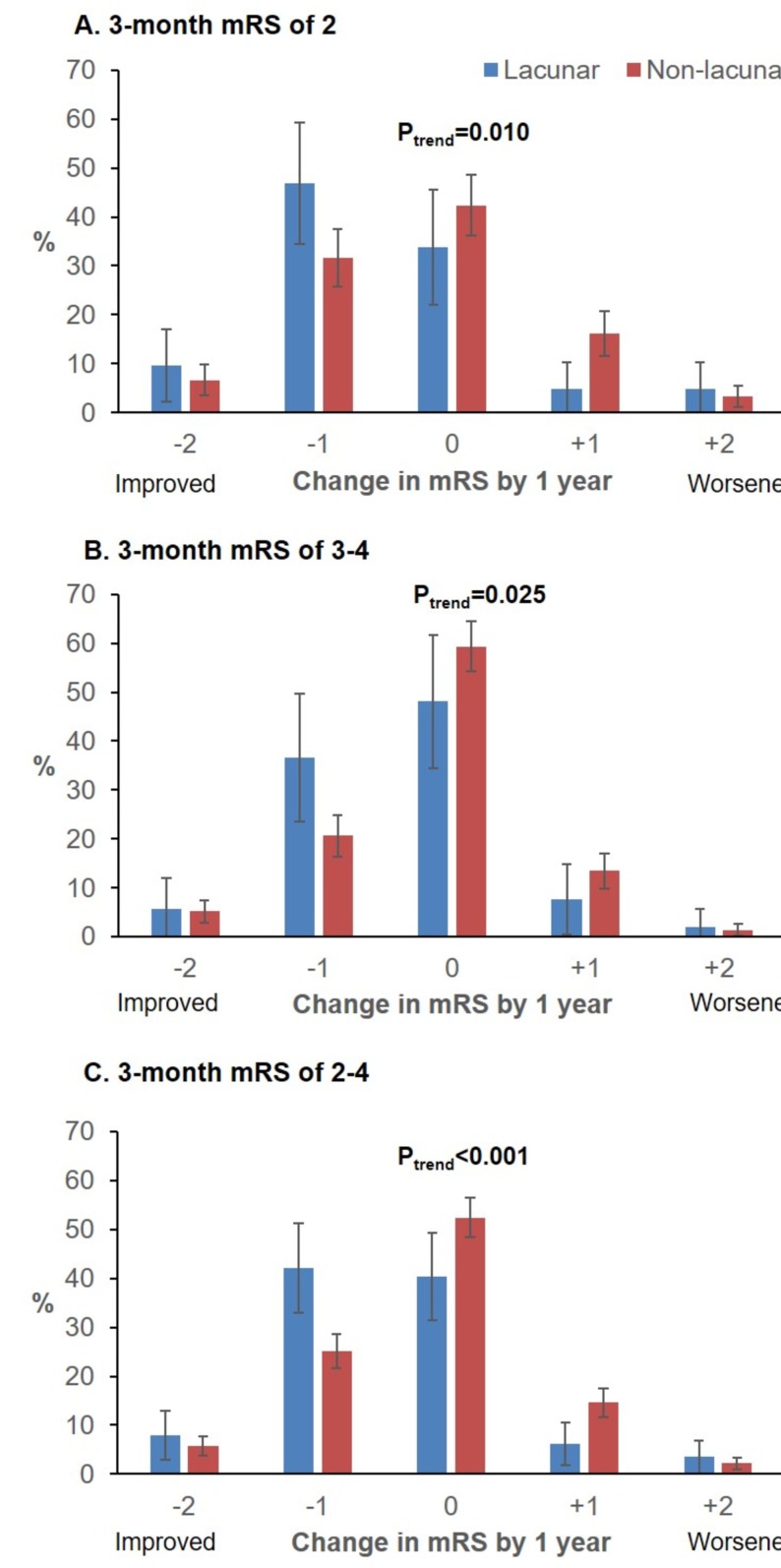
Changes in modified Rankin Scale (mRS) between 3-months and 1-year post-stroke for 3-month survivors of lacunar versus non-lacunar strokes with 3-month mRS of 2, 3, 4, and 2 to 4 (pooled), potentially recruitable for trials of recovery therapies. On the x-axis, 0 indicates no change in mRS, positive points to the right indicate mRS worsening, while negative points to the left indicate improvement. Results of non-parametric tests for trend significance (P trend) are shown above. Results for 3-month mRS 3-4 were combined because there were only 15 lacunar strokes with 3-month mRS=4.

**Figure 2:**
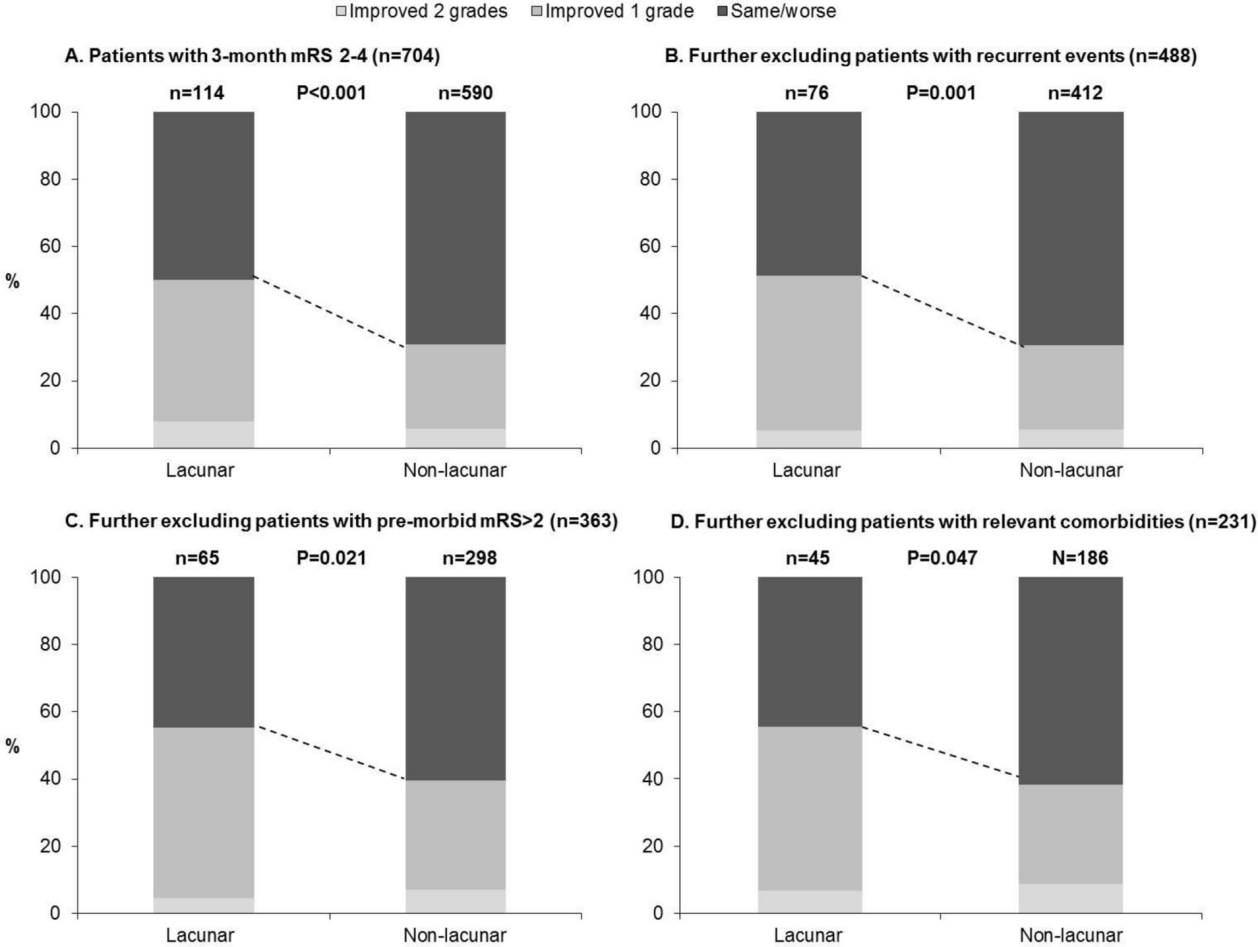
Changes in mRS between 3-months and 1-year post-stroke for 3-month survivors of lacunar versus non-lacunar stroke, (A) including all patients with 3-month mRS of 2 to 4, and then progressively excluding patients with: (B) recurrent vascular events over follow-up, (C) pre-morbid mRS>2, and (D) relevant comorbidities including peripheral vascular disease, heart failure, valve disease, and/or cancer. P-values are from Wilcoxon rank-sum tests for trend.

Patients with lacunar strokes were, however, much more likely to show improvement by ≥1 mRS grades between 3 months and 6 months (71/214 (33.2%) vs 242/1052 (23.0%); 3-month mRS 2–4: 49/114 (43.0%) vs 160/621 (25.8%), p<0.001; online supplementary figure VI), even adjusting for age/sex/3-month mRS (aOR: 1.55, 1.11 to 2.18, p=0.01, n=1206). These patients were also more likely to demonstrate improvement between 6 months and 1 year (39/198 (19.7%) vs 139/973 (14.3%); 3-month mRS 2–4: 24/96 (25.0%) vs 87/557 (15.6%), p=0.02; online supplementary figure VII). Consequently, improvement between 3 months and 1 year was also more common for lacunar strokes (75/214 (35.1%) vs 251/992 (25.3%), aOR: 1.64, 1.17 to 2.31, p=0.004, n=1206; table 2 and online supplementary figure VIII). This difference remained on examining only those with 3-month mRS of 2–4 (57/114 (50.0%), 182/590 (30.9%), p <0.001, figure 1), excluding recurrent events (aOR: 2.28, 1.34 to 3.86, p=0.002, n=488), adjusting for premorbid disability (aOR: 2.11, 1.23 to 3.61, p=0.007), and further excluding patients with premorbid disability or relevant chronic conditions (figure 2).

**Table 2:** Logistic regression models for the association of lacunar versus non-lacunar stroke with functional improvement per mRS, RMI and/or BI between 3 months and 1-year poststroke, adjusted for age, sex and 3-month score on the relevant measure, in 3-month survivors of ischaemic stroke

	mRS improvement between 3 months and 1 year		RMI improvement between 3 months and 1 year		BI and/or RMI improvement between 3 months and 1 year		mRS, RMI and/or BI improvement between 3 months and 1 year	
	aOR (95% CI)	p>|z|	aOR (95% CI)	p>|z|	aOR (95% CI)	p>|z|	aOR (95% CI)	p>|z|
Lacunar stroke (vs non-lacunar)	1.64 (1.17 to 2.31)	0.004	1.78 (1.20 to 2.64)	0.004	1.55 (1.06 to 2.26)	0.024	1.70 (1.26 to 2.28)	0.001
Age	0.97 (0.96 to 0.98)	<0.0001	0.97 (0.95 to 0.98)	<0.0001	0.98 (0.97 to 0.99)	0.001	0.99 (0.98 to 1.00)	0.083
Male	1.26 (0.96 to 1.66)	0.096	1.27 (0.94 to 1.71)	0.123	1.10 (0.83 to 1.46)	0.52	0.98 (0.78 to 1.23)	0.864
3-month mRS	1=reference		NA		NA		NA	
2	4.68 (3.25 to 6.73)	<0.0001						
3	2.60 (1.71 to 3.97)	<0.0001						
4	4.03 (2.57 to 6.34)	<0.0001						
5	2.90 (1.59 to 5.27)	<0.0001						
3-month RMI	NA		0.96 (0.93 to 0.99)	0.028	NA		NA	
3-month BI	NA		NA		0.97 (0.95 to 1.00)	0.042		
Initial NIHSS	NA		NA		NA		1.02 (1.00 to 1.05)	0.040
	p>|X^2^| n	<0.0001 1206	p>|X^2^| n	<0.0001 752	p>|X^2^| n	<0.0001 799	p>|X^2^| n	0.001 1251

For the model examining improvement in any of the three scales, we adjusted for the initial stroke severity (NIHSS score). These models exclude patients who could not show improvement by definition, namely those with 3-month mRS=0 (n=137), 3-month RMI=15 (n=378) or 3-month BI=20 (n=674), with 93 patients meeting all three criteria.

aOR, adjusted OR; BI, Barthel Index; mRS, modified Rankin Scale; NA, not applicable; NIHSS, National Institutes of Health Stroke Scale; RMI, Rivermead Mobility Index.

Similar trends were observed for functional improvement per RMI/BI between 3 months and 1 year (online supplementary figure IX). Of 225 patients with 3-month mRS ≥1 and 3-month RMI <15 or 3-month BI <20 whose mRS score improved between 3 months and 1 year, 161 (71.6%) also showed an improvement in the RMI and/or BI (OR for patients with improved mRS also showing improved RMI/BI: 3.44, 2.46 to 4.80, p<0.0001; BI: 7.50, 4.81 to 11.7, p<0.0001). Patients with lacunar strokes were also significantly more likely to show improvement on RMI/BI between 3 months and 1 year, adjusted for age/sex/3-month RMI/BI (aOR: 1.55, 1.06 to 2.26, p=0.02, n=799; table 2). This difference remained on adjusting for recurrent events and premorbid BI (aOR: 1.59, 1.09 to 2.33, p=0.02; aOR (RMI): 1.80, 1.21 to 2.67, p=0.004) or excluding patients with recurrent events and/or premorbid disability (online supplementary figures X, XI). Patients with lacunar stroke were also more likely to improve between 3 months and 1 year on ≥1 of the three scales (mRS/BI/RMI) adjusted for age/sex/NIHSS score (aOR: 1.70, 1.26 to 2.28, p=0.001, n=1251; table 2), even on restricting to patients with 3-month mRS of 2–4 and excluding those with recurrent events (aOR: 2.32, 1.34 to 4.02, p=0.003, n=488) .

Beyond 1 year, improvements on mRS were rarer in both groups (lacunar: 21/179 (11.7%), rest: 93/787 (11.8%), p=0.98; online supplementary figure XII) and no more likely for patients with lacunar strokes after adjusting for age/sex/1-year mRS (aOR (mRS): 0.84, 0.50 to 1.43, p=0.53, n=961, excluding deaths/recurrent events: 0.92, 0.51 to 1.69, p=0.80, n=484). Similarly, patients with lacunar stroke were no more likely than other 5-year survivors to show further improvement on RMI/BI beyond 1 year (aOR: 0.83, 0.46 to 1.47, p=0.51, n=590, excluding deaths/recurrent events: 0.77, 0.38 to 1.56, p=0.47, n=242; online supplementary figure XIII).

Similar results were seen when performing these analyses with patients with LACS versus other syndromes, despite the greater similarity of their baseline characteristics (online supplementary table I and figure XIV). Patients with LACS strokes were no more likely to show early functional improvement within 3 months (aOR for 3-month mRS >2: 1.26, 0.89 to 1.77, p=0.19), but were significantly more likely to show late improvement between 3 months and 1 year (aOR (mRS): 1.37, 1.02 to 1.84, p=0.03), particularly between 3 months and 6 months (aOR: 1.62, 1.12 to 2.33, p=0.01, excluding recurrent events and prestroke mRS >2). Similarly, 1-year survivors were more likely to show improvement on RMI/BI between 3 months and 1 year, adjusted for age/sex/3-month RMI/BI/recurrent events (aOR (RMI/BI): 1.44, 1.03 to 2.01, p=0.03; aOR (RMI): 1.41, 1.01 to 1.98, p=0.04, n=760). Beyond 1 year, no differences in functional improvement were seen (aOR (mRS): 0.90, 0.66 to 1.24, p=0.53; aOR (RMI/BI): 0.72, 0.38 to 1.37, p=0.32).

## Discussion

By prospective assessment of disability using three commonly used scales in a population-based cohort study, we showed that patients with lacunar strokes have greater potential for late functional improvement in the first year poststroke. This difference remained significant in multiple sensitivity analyses and was not accounted for by differences in 3-month disability, premorbid or non-stroke-related disability, mortality, or recurrent events. Functional improvement beyond 1 year was rare and no more likely in patients with lacunar stroke. Our findings have implications for motivating and rehabilitating patients with lacunar stroke in clinical practice, for the design of restorative therapy trials, and for our understanding of functional recovery.

First, our findings should encourage clinicians to optimise late functional improvement in patients with lacunar stroke, and to consider this added potential for late improvement when discussing prognosis and rehabilitation options. These findings could be interpreted in support of piloting and focusing studies of non-acute stroke restorative therapies in patients with lacunar stroke. In addition to the lower mortality of lacunar strokes,^28^ their greater potential for late functional improvement makes them especially appealing for enrolment in studies of new therapies. Such patient selection might improve detection of treatment effects that could be missed in a mixed sample of non-lacunar or cortical strokes. For example, analyses of a robot-based rehabilitation study^29^ and a neutral trial of epidural motor cortex stimulation^30^ found that responders were typically lacunar/subcortical strokes with intact motor system physiology, specifically cortical function and connectivity. On the other hand, the relatively lower rate of late functional improvement in patients with non-lacunar stroke in our cohort does not mean that interventions to improve recovery in this group are futile. One might also argue that if patients with lacunar stroke are likely to demonstrate late improvement without additional therapy, then attempts at restorative therapies may be better concentrated on patients with non-lacunar stroke whose functional improvements might otherwise plateau. Indeed, the apparent differences in potential for late functional improvement between patients with lacunar and non-lacunar strokes may reflect differences in engagement in rehabilitation; although both groups appeared to receive similar levels of inpatient and outpatient rehabilitation, patients with lacunar strokes may have engaged more effectively or aggressively in these sessions owing to factors like more intact cortex and/or lower cognitive impairment.

Second, our findings imply that studies of restorative therapies cannot assume that functional improvements seen in the first year poststroke are necessarily treatment-related, even if undertaken beyond 3 months poststroke. Studies focusing on patients with lacunar strokes should be randomised and controlled to ensure that improvement exceeds what is expected from their natural history. For instance, some early uncontrolled studies of repetitive transcranial magnetric stimulation have reported that patients with lacunar/subcortical strokes were most likely to improve, but this difference cannot necessarily be ascribed to a treatment response.^31^ Similarly, if such therapies are tested in the general stroke population, the treatment and control groups should be balanced in their representation of lacunar strokes to prevent confounding by a greater capacity for late improvement in either group.

Third, our findings lend credence to the phenomenon of late recovery beyond 3 months poststroke and underscore the importance of further studying mechanisms of late subcortical or white matter recovery. Emerging evidence indicates that these mechanisms include time-dependent processes like cortical activation, network modulation,^32^ contralesional cortical reorganisation,^33^ enhanced interhemispheric connectivity,^34^ and modulation of axomyelinic synapses to alter myelin properties or recruit companion glia.^35^ MRI lesions shrink over 1 year in almost half of patients with lacunar strokes,^36^ with axonal remodelling giving rise to poorly organised, randomly oriented axons in the initial poststroke months, followed by gradual organisation into single direction-oriented fibres.^37^ These mechanisms may explain why our patients with lacunar stroke showed no difference in 3-month functional improvement rates versus non-lacunar stroke patients, but were more likely to show further improvement over the next 9 months. That these differences persisted on adjusting for age is compatible with recent evidence that white matter neuroplasticity, unlike cortical plasticity, does not diminish with age.^38^

Although our analysis has several strengths, including generalisability from a population-based sample, there are some shortcomings. First, we assessed functional improvement using disability scales and did not serially determine neurological impairments using measures like the NIHSS or Fugl-Meyer Scale, which may be more sensitive to small improvements in deficits.^39^ However, any insensitivity would likely cause similar underestimation of improvement in patients with lacunar and non-lacunar strokes, and a small improvement on an impairment-base scale may not translate into meaningful functional improvement in the patient’s daily activities. On the other hand, our use of functional outcome scales means that we cannot differentiate between improvement in neurological impairment and adaptation to impairment, as either of these processes can result in functional improvement. Additional studies using serial measurements of neurological impairment will therefore be required to further clarify the nature of this observed late functional improvement in lacunar strokes. Second, scales like the mRS can be confounded by non-stroke-related disability. However, between-group differences remained significant after adjusting for and/or excluding premorbid disability and excluding those with potentially disabling comorbidities. Third, the mRS and other scales have inter-rater variability,^20^ but our findings were similar for the mRS and for the BI and RMI, and inter-rater variability would be unlikely to account for differences between lacunar and non-lacunar stroke. Fourth, we coded functional improvement as a binary outcome in logistic regressions for our main adjusted analyses; more sophisticated models of mRS/RMI/BI changes over time, such as multilevel models with random intercepts to account for repeated measures for each patient, may have better captured differences in the extent of improvement between patients with lacunar and non-lacunar strokes. However, we did examine functional improvement as a scalar outcome in univariate analyses (presented in the graphs), which while unadjusted were grouped by relevant parameters like 3-month mRS and age, and demonstrated significant differences between patients with lacunar and non-lacunar strokes as with the logistic regressions. Finally, we could not adjust for all psychosocial factors that might affect functional improvement, such as mood/anxiety, social support and economic status. However, we suspect that such interindividual variability is unlikely to have driven the between-group differences in our study.

In conclusion, patients with lacunar strokes have greater potential for late functional improvement in the first year poststroke, which should motivate patients and clinicians to maximise late improvements in routine practice. However, since late recovery is common, studies of restorative therapies that enrol patients with lacunar strokes should be randomised and controlled to reliably assess treatment effects. More detailed studies of late recovery of specific neurological deficits might help elucidate the nature of this late improvement.
