# Supplementary material for: Late functional improvement after lacunar stroke: a population-based study
Source: J Neurol Neurosurg Psychiatry. 2018 Jul 21;89(12):1301–7. doi: 10.1136/jnnp-2018-318434 (PMC6288699; doi:10.1136/jnnp-2018-318434)
Supplement: Supplementary data [file jnnp-2018-318434supp001.pdf]

ONLINE SUPPLEMENT

Supplemental Tables

Table I. Patient sample and characteristics for 3-month survivors of ischaemic stroke, classified per Oxfordshire Community Stroke Project criteria for lacunar stroke syndromes (LACS)

**Characteristic**	**All strokes** (n=1,425)	**LACS strokes** (n=362)	**Non-LACS strokes** (n=1,063)	**Age- and sex-adjusted P**
**Age, mean (S.D.)**	73.2 (12.7)	71.5 (12.6)	73.7 (12.6)	
**Sex – male (%)**	753 (52.8)	207 (57.2)	541 (50.9)	
**Previous history(%):**				
MI	177 (12.4)	35 (9.7)	141 (13.3)	0.07
Angina	239 (16.8)	58 (16.0)	177 (16.7)	0.33
Atrial Fibrillation	260 (18.2)	38 (10.5)	214 (20.1)	0.87
Hypertension	889 (62.4)	225 (62.2)	653 (61.4)	0.65
Dyslipidemia	469 (32.9)	108 (29.8)	358 (33.7)	0.10
Diabetes	205 (14.4)	56 (15.5)	146 (13.7)	0.56
PVD	108 (7.6)	25 (6.9)	83 (7.8)	0.52
Stroke	158 (11.1)	39 (10.8)	114 (10.7)	0.76
TIA	205 (14.4)	48 (13.3)	155 (14.6)	0.66
Smoking	836 (58.7)	224 (61.9)	608 (57.2)	0.60
Heart Failure	119 (8.4)	24 (6.6)	91 (8.6)	0.36
Valvular Heart Disease	134 (9.4)	27 (7.5)	104 (9.8)	0.26
Cancer	22 (15.5)	57 (15.8)	159 (15.0)	0.44
Pre-stroke mRS >2	244 (17.2)	47 (13.0)	185 (17.4)	0.24
Pre-stroke BI <20	318 (22.3)	72 (19.9)	233 (21.9)	0.86
**Initial NIHSS**, mean (SD)	3.7 (5.0)	2.6 (2.8)	4.2 (5.6)	**<0.001***
**Recurrent stroke** within 5-years of follow-up (%)	211 (14.8)	47 (13.0)	164 (15.4)	0.40
**Any recurrent vascular event** within 5-years (%)	360 (25.3)	88 (24.3)	272 (25.6)	0.81
**Post-stroke depression** (%)	350 (24.6)	96 (26.5)	254 (23.9)	0.32
**Deaths** (%)				
Within 1-year	138 (9.7)	19 (5.3)	119 (11.2)	**0.007***
Within 5-years	464 (37.1)	91 (28.1)	374 (40.3)	**0.01***

Significant differences (p<0.05) between lacunar and non-lacunar strokes are indicated by an asterisk (*). Age was compared using an independent samples T-test, sex using the Chi-squared test, NIHSS using age/sex-adjusted linear regression, and the rest using age/sex-adjusted logistic regression. Abbreviations: AF – Atrial Fibrillation, S.D. – Standard deviation, MI – Myocardial Infarction, PVD – Peripheral Vascular Disease, TIA – Transient Ischaemic Attack, mRS – modified Rankin scale, BI – Barthel Index, NIHSS – National Institutes of Health Stroke Scale.

Supplemental Figures

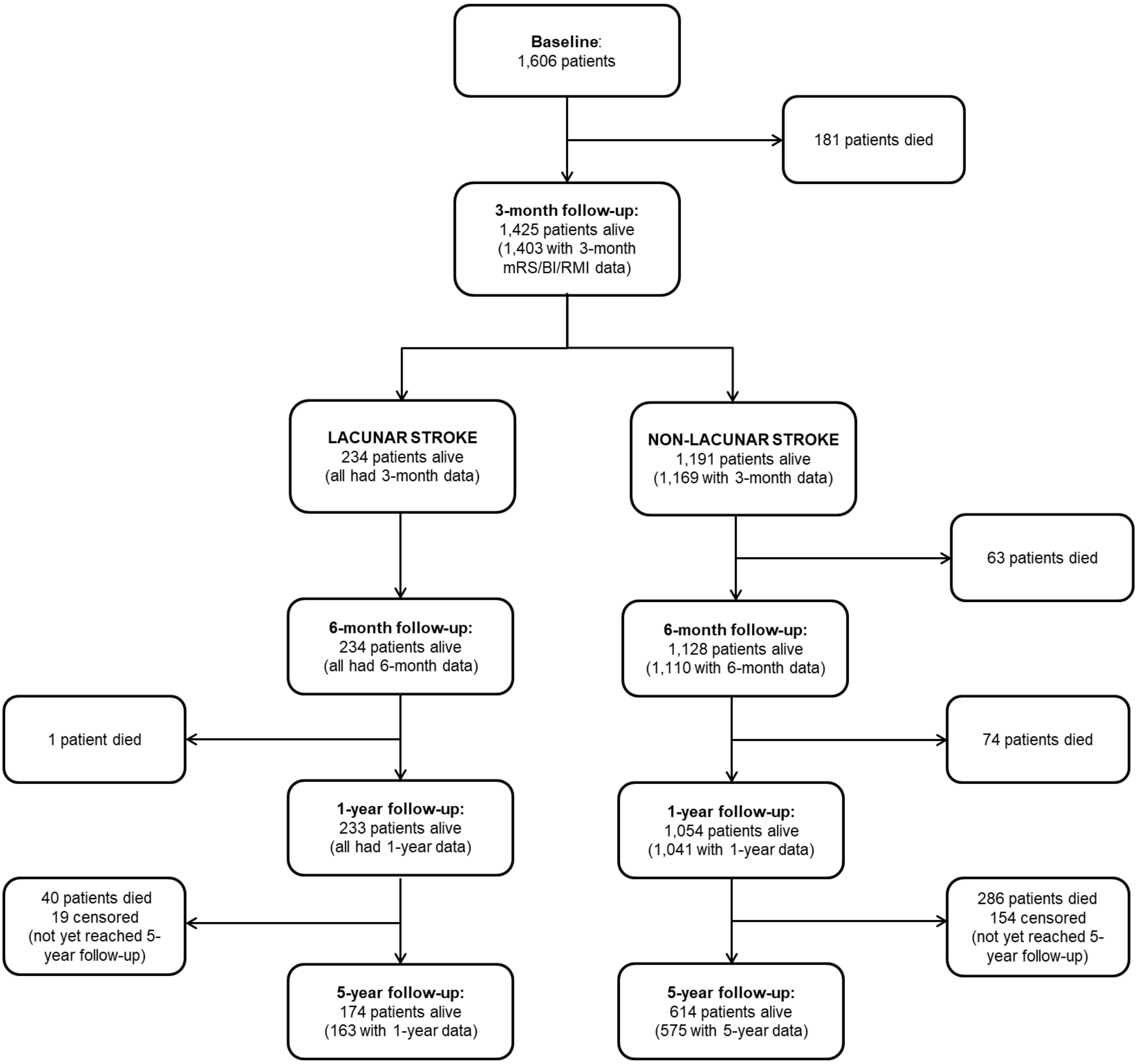

Figure I - Flowchart illustrating the ischaemic stroke patients with lacunar and non-lacunar strokes who were alive at baseline and at 3-month, 6-month, 1-year, and 5-year follow-up assessments. The number of patients with available follow-up data at each time-point are also indicated.

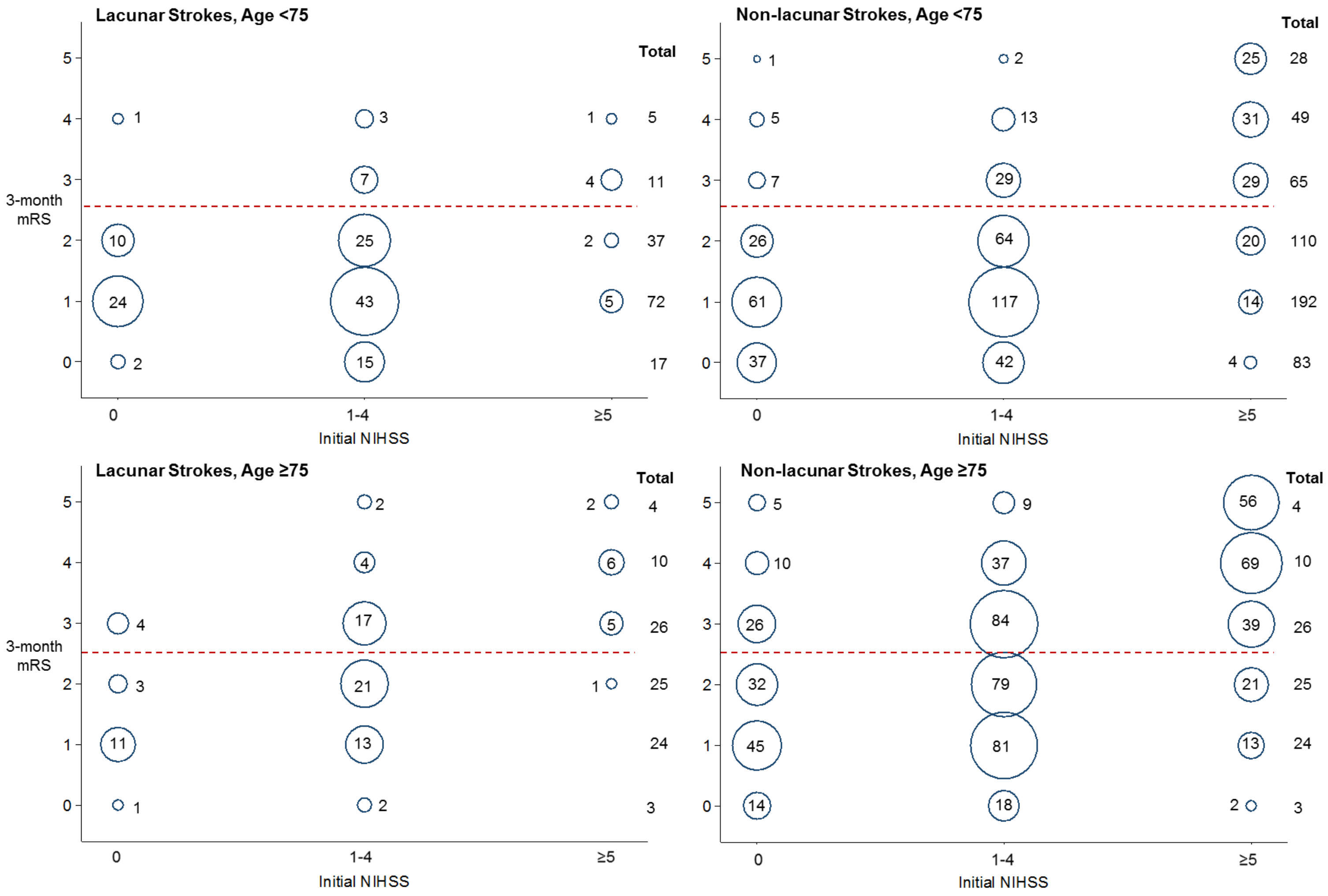

Figure II. 3-month mRS scores by initial NIHSS score, stratified by age<75 and ≥75, for 3-month survivors with lacunar strokes and non-lacunar strokes. The size of each bubble represents the number of patients at that intersection. The dashed line separates patients with moderate or higher disability (mRS 3-5) from those with no or mild disability (mRS 0-2).

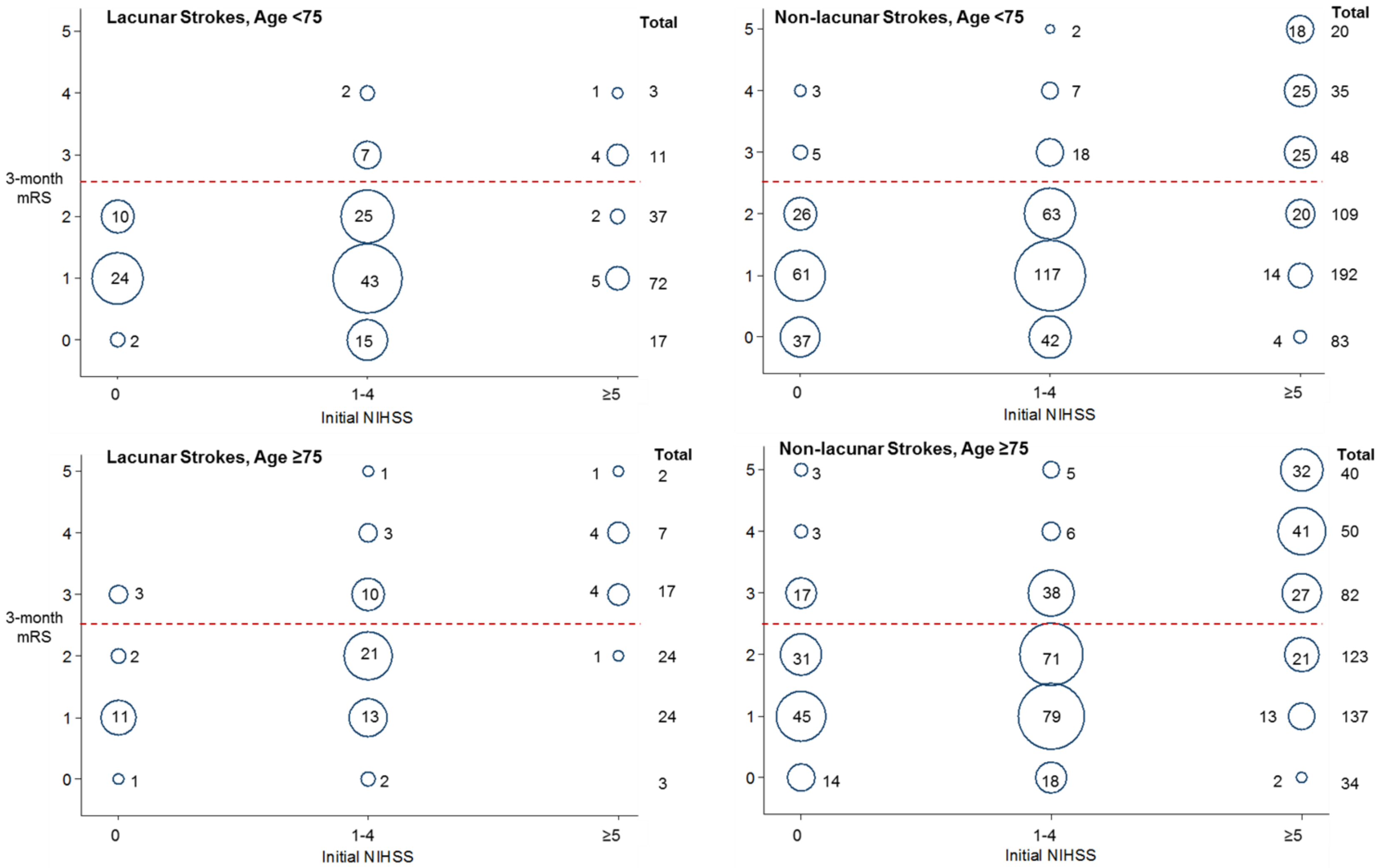

Figure III. 3-month mRS scores by initial NIHSS score, stratified by age<75 and ≥75, for 3-month survivors with lacunar strokes and non-lacunar strokes, excluding those with pre-stroke mRS>2. The size of each bubble represents the number of patients at that intersection. The dashed line separates patients with moderate or higher disability (mRS 3-5) from those with no or mild disability.

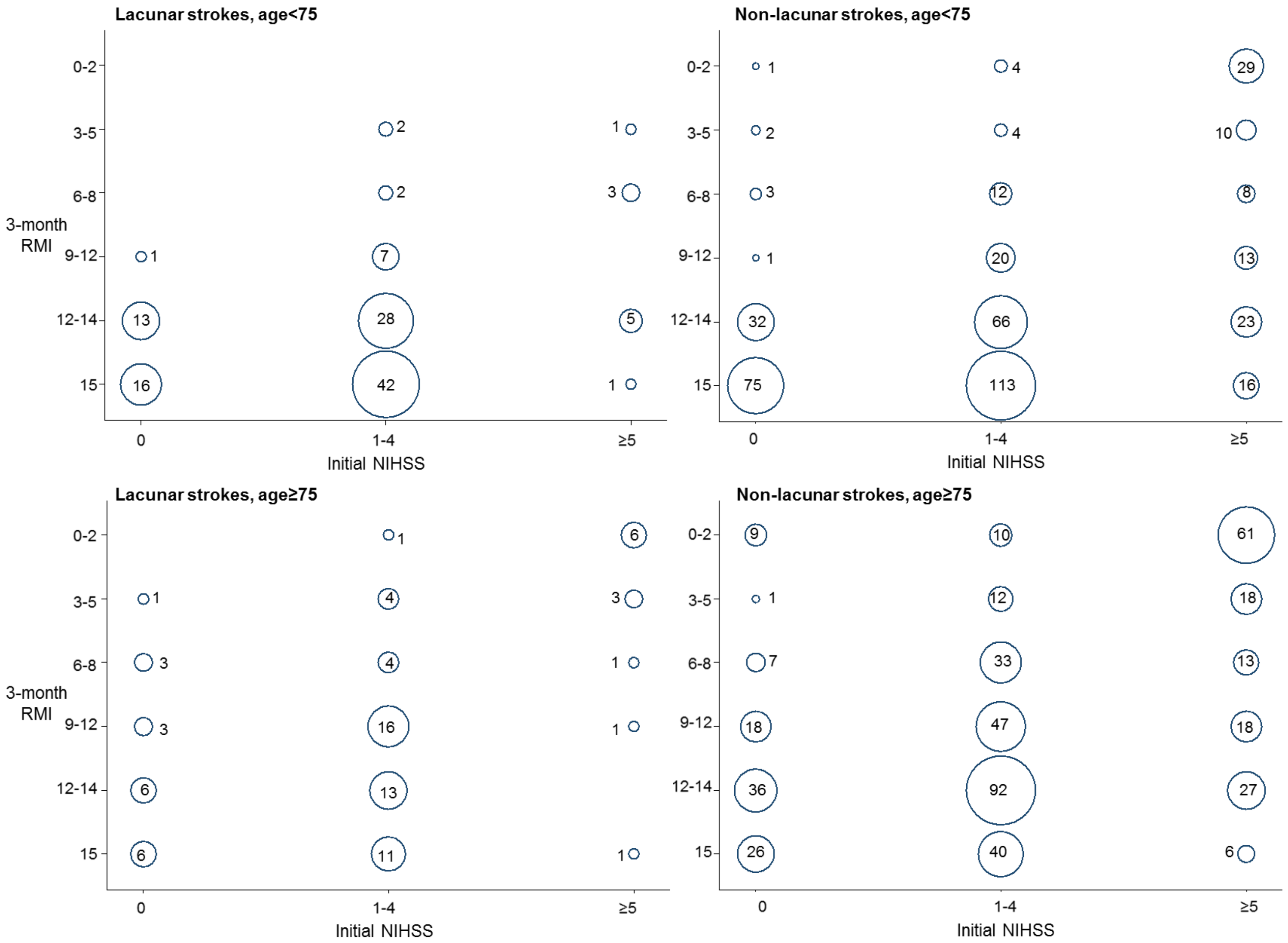

Figure IV. 3-month Rivermead Mobility Index (RMI) scores by initial NIHSS score, stratified by age<75 and ≥75, for 3-month survivors with lacunar strokes and non-lacunar strokes.

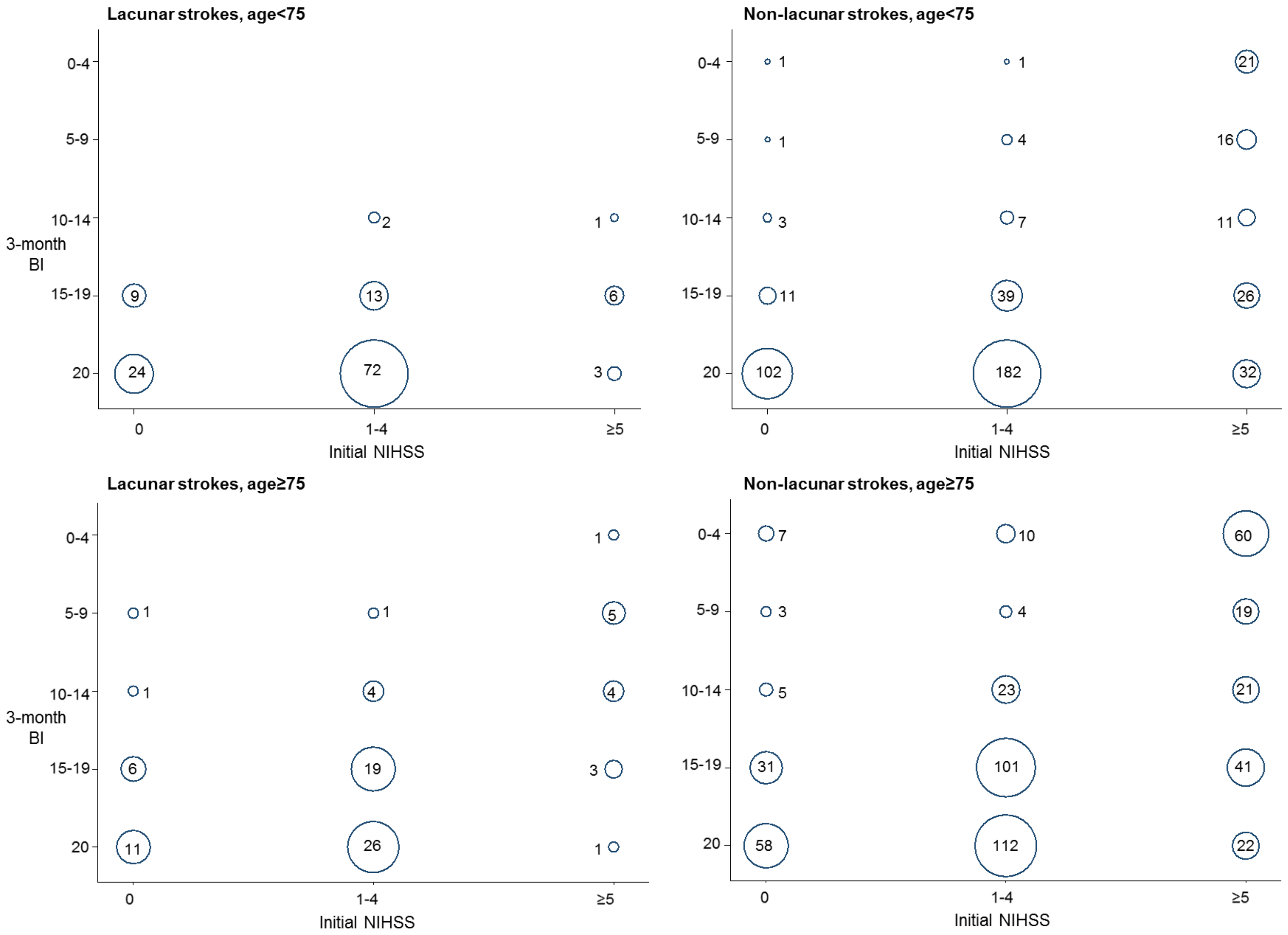

Figure V. 3-month Barthel Index (BI) scores by initial NIHSS score, stratified by age<75 and ≥75, for 3-month survivors with lacunar strokes and non-lacunar strokes.

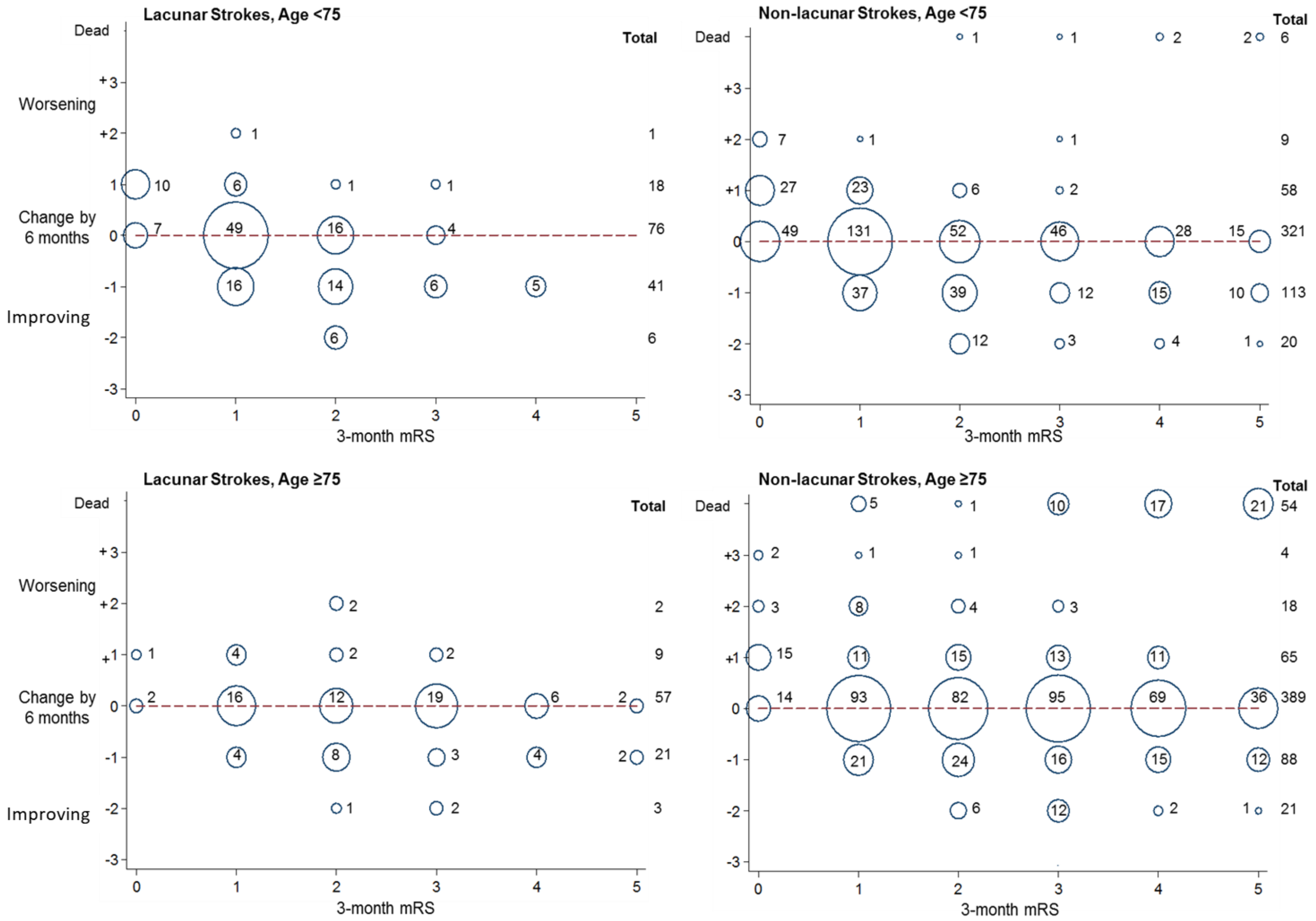

Figure VI. Changes in mRS between 3-months and 6-months post-stroke for 3-month survivors with lacunar and non-lacunar stroke, stratified by age<75 and ≥75. The size of each bubble represents the number of patients at that intersection. Bubbles falling along the dashed line indicate no change in mRS. Bubbles falling above this line indicate a worsening of mRS score in that time period, while those falling below it indicate improvement.

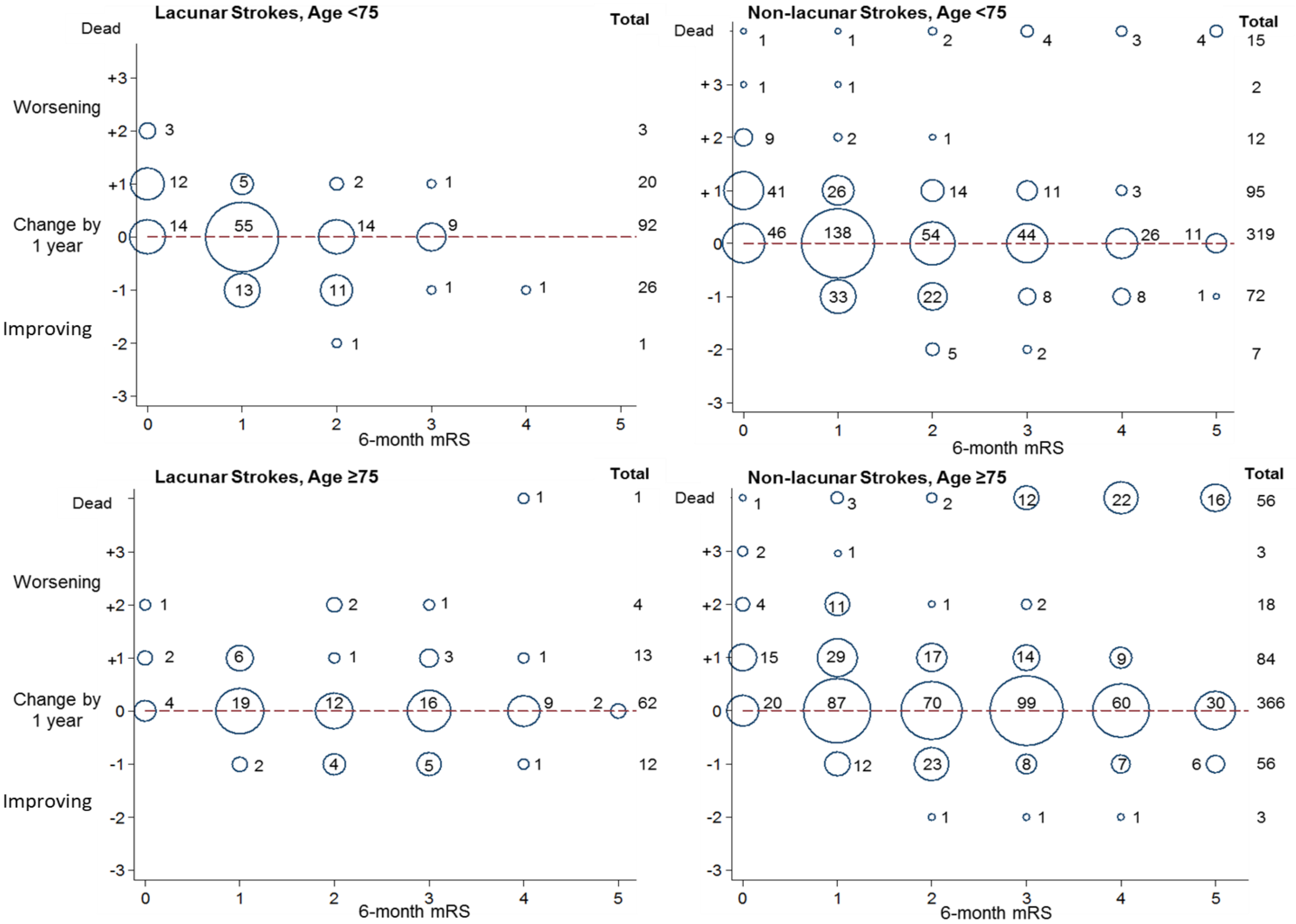

Figure VII. Changes in mRS between 6-months and 1-year post-stroke for 6-month survivors with lacunar and non-lacunar stroke, stratified by age<75 and ≥75. The size of each bubble represents the number of patients at that intersection. Bubbles falling along the dashed line indicate no change in mRS. Bubbles falling above this line indicate a worsening of mRS score in that time period, while those falling below it indicate improvement.

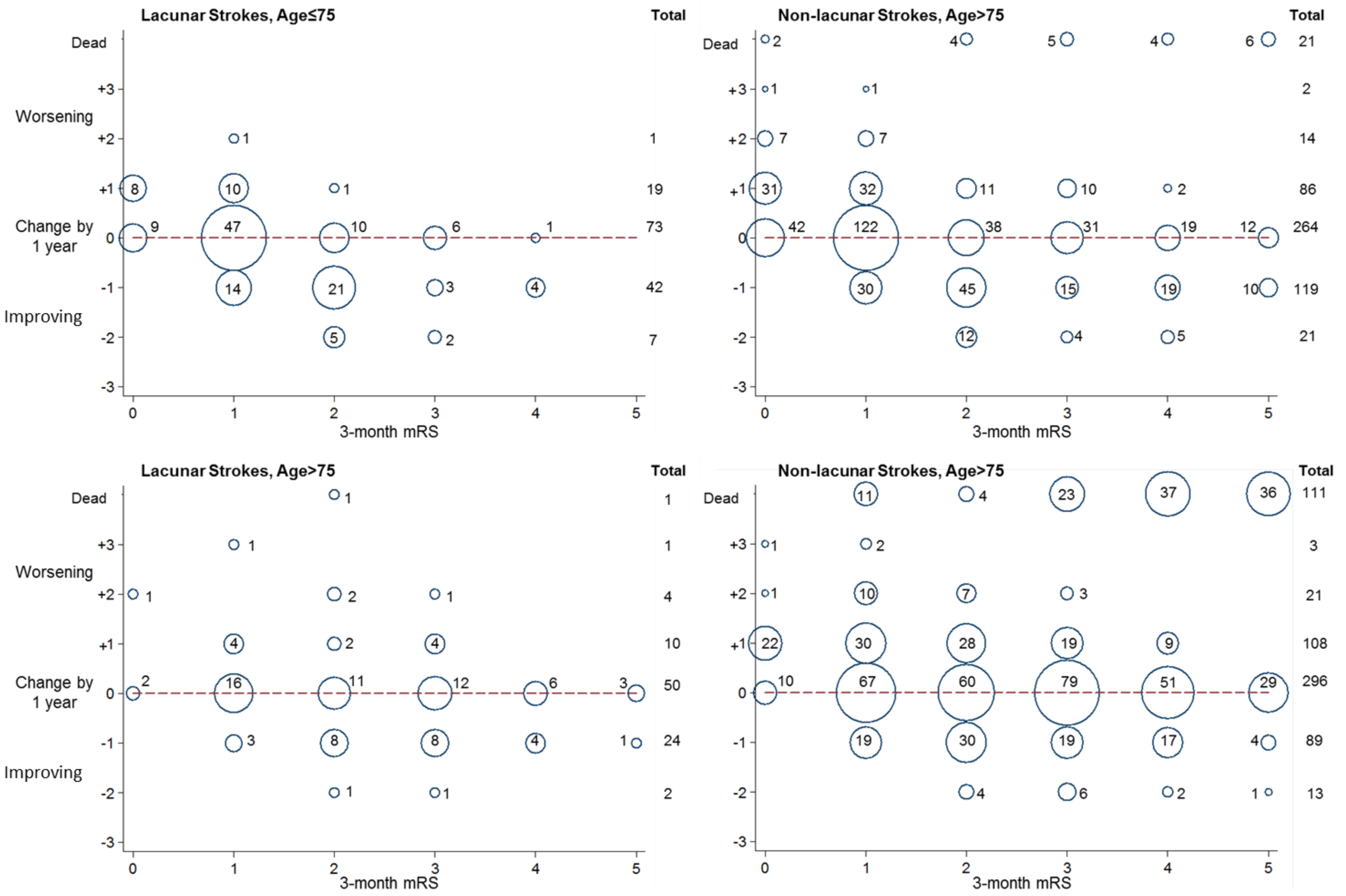

Figure VIII. Changes in mRS between 3-months and 1-year post-stroke for 3-month survivors with lacunar and non-lacunar stroke, stratified by age<75 and ≥75. The size of each bubble represents the number of patients at that intersection. Bubbles falling along the dashed line indicate no change in mRS. Bubbles falling above this line indicate a worsening of mRS score in that time period, while those falling below it indicate improvement.

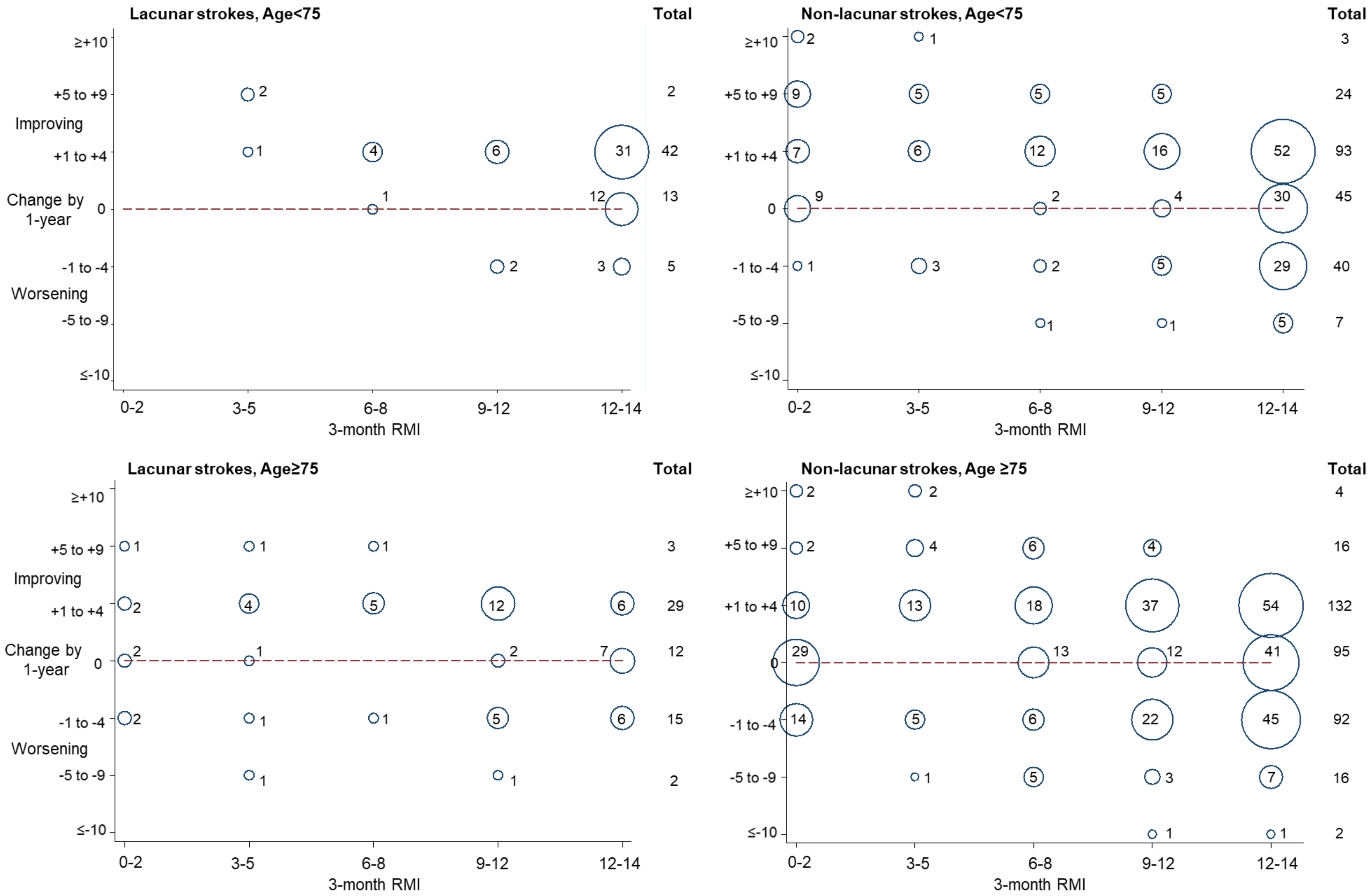

Figure IX. Changes in RMI between 3-months and 1-year post-stroke for 1-year survivors with lacunar and non-lacunar stroke and a 3-month RMI<15, stratified by age<75 and ≥75. The size of each bubble represents the number of patients at that intersection. Bubbles falling along the dashed line indicate no change in RMI. Bubbles falling below this line indicate a worsening of RMI score in that time period, while those falling above it indicate improvement.

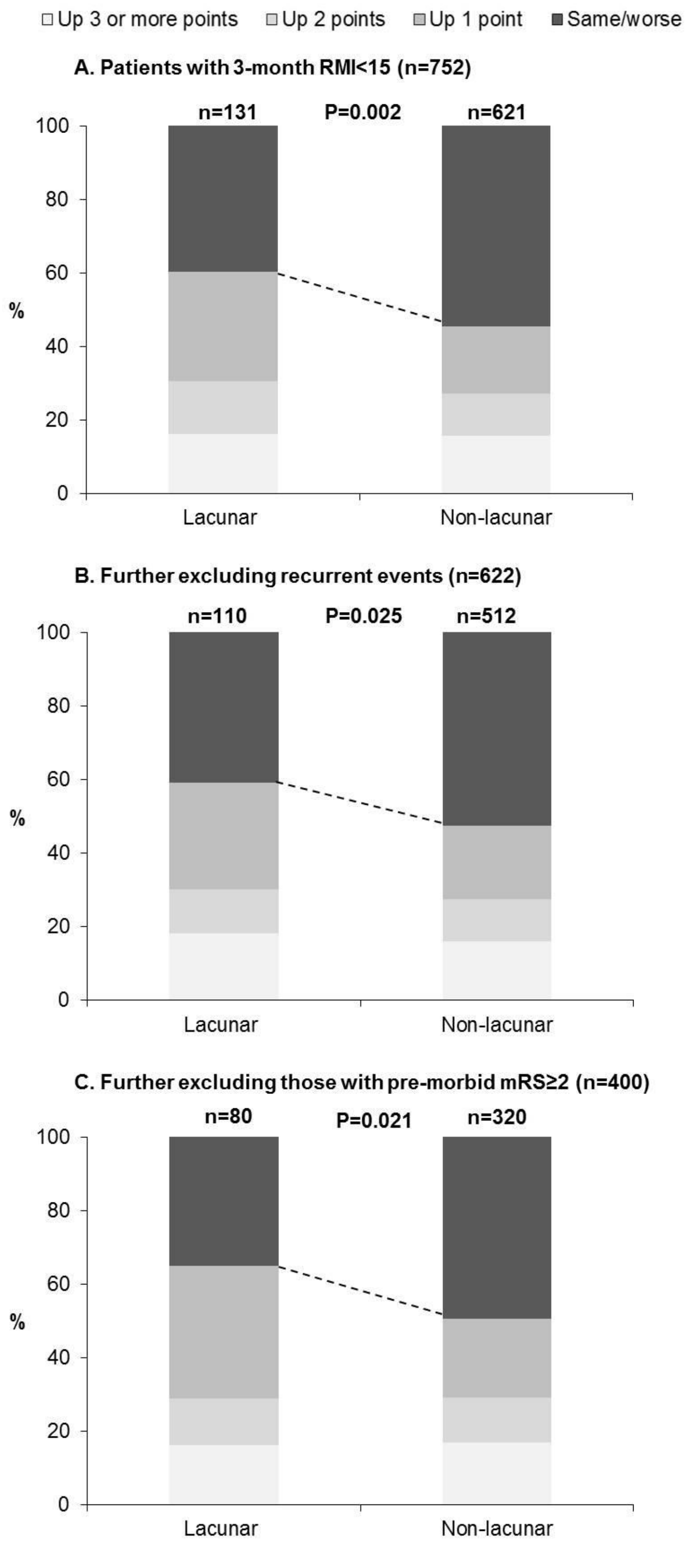

Figure X**. Changes in Rivermead Mobility Index(RMI) between 3-months and 1-year post-stroke for 3-month survivors of lacunar versus non-lacunar stroke**, (A) including all patients with 3-month RMI<15, then progressively excluding those with: (B) recurrent strokes over follow-up, and (C) pre-morbid mRS≥2. P-values are from Wilcoxon rank-sum tests for trend.

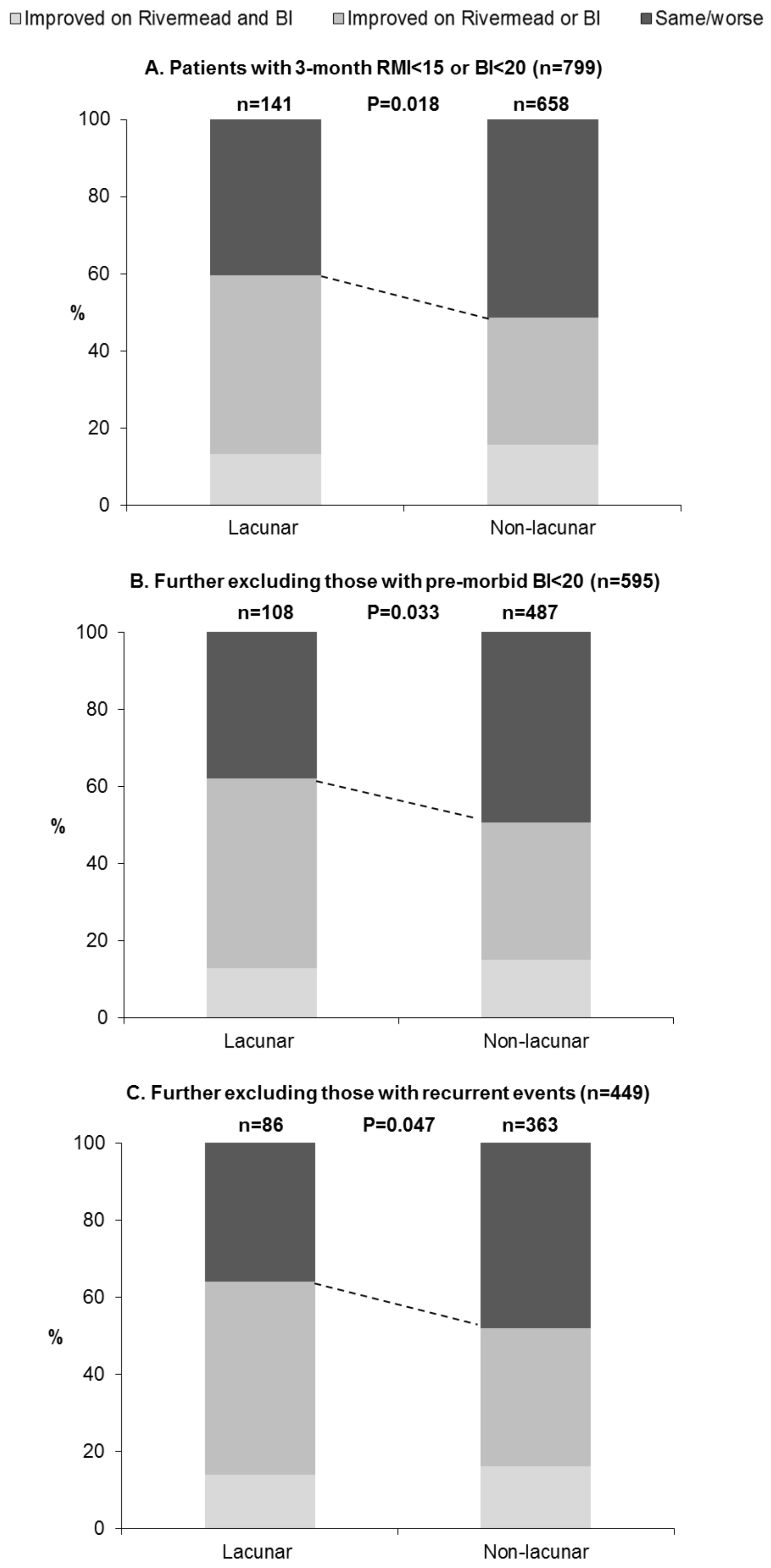

Figure XI. Changes in Rivermead Mobility Index (RMI) and/or Barthel Index (BI) between 3-months and 1-year post-stroke for 3-month survivors of lacunar versus non-lacunar stroke, (A) including all patients with 3-month RMI<15, and then progressively excluding those with (B) pre-morbid BI<20 and (C) recurrent vascular events over follow-up. P-values shown are from Wilcoxon rank-sum tests.

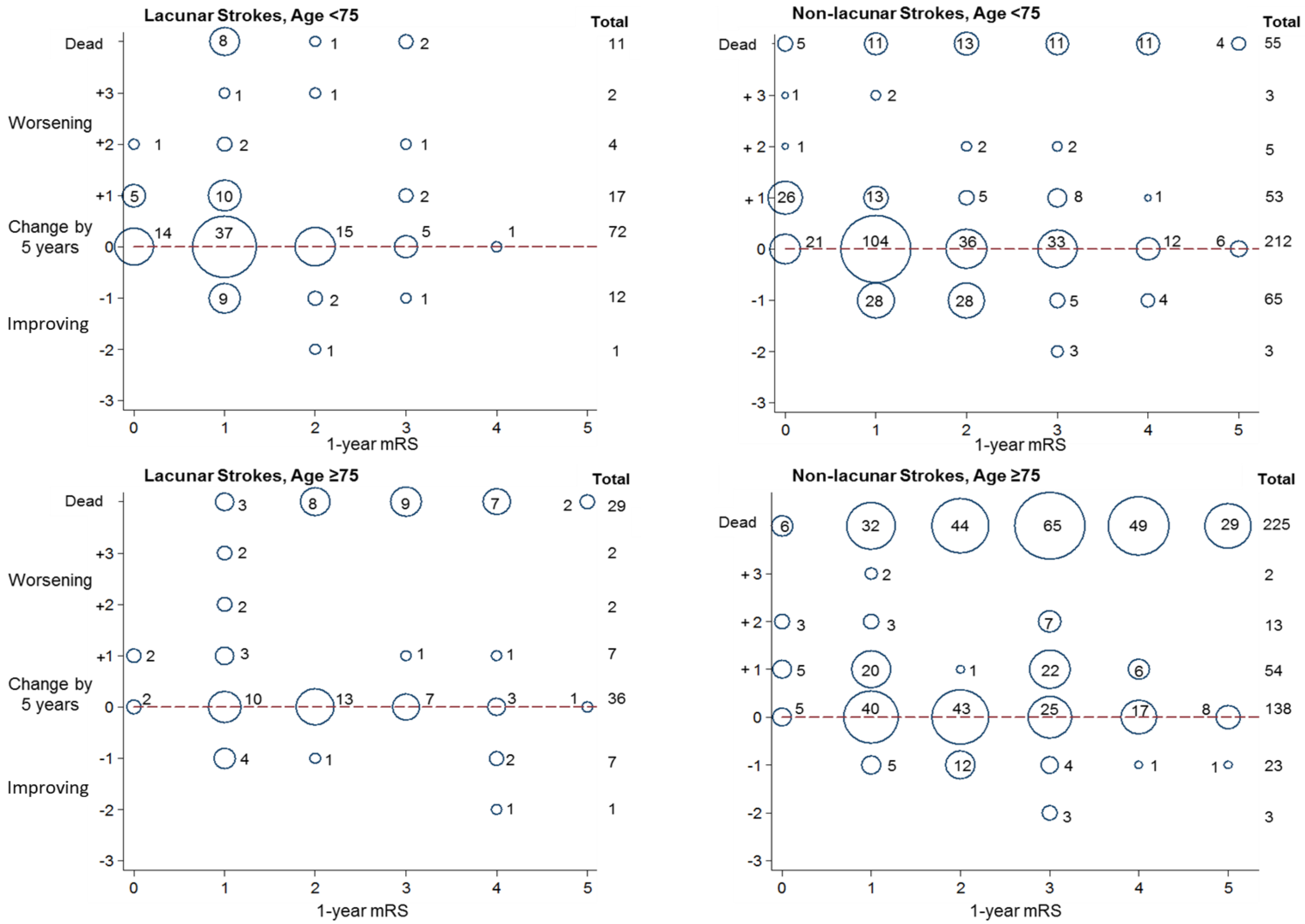

Figure XII. Changes in mRS between 1-year and 5-years post-stroke for 1-year survivors with lacunar and non-lacunar stroke, stratified by age<75 and ≥75. The size of each bubble represents the number of patients at that intersection. Bubbles falling along the dashed line indicate no change in mRS. Bubbles falling above this line indicate a worsening of mRS score in that time period, while those falling below it indicate improvement.

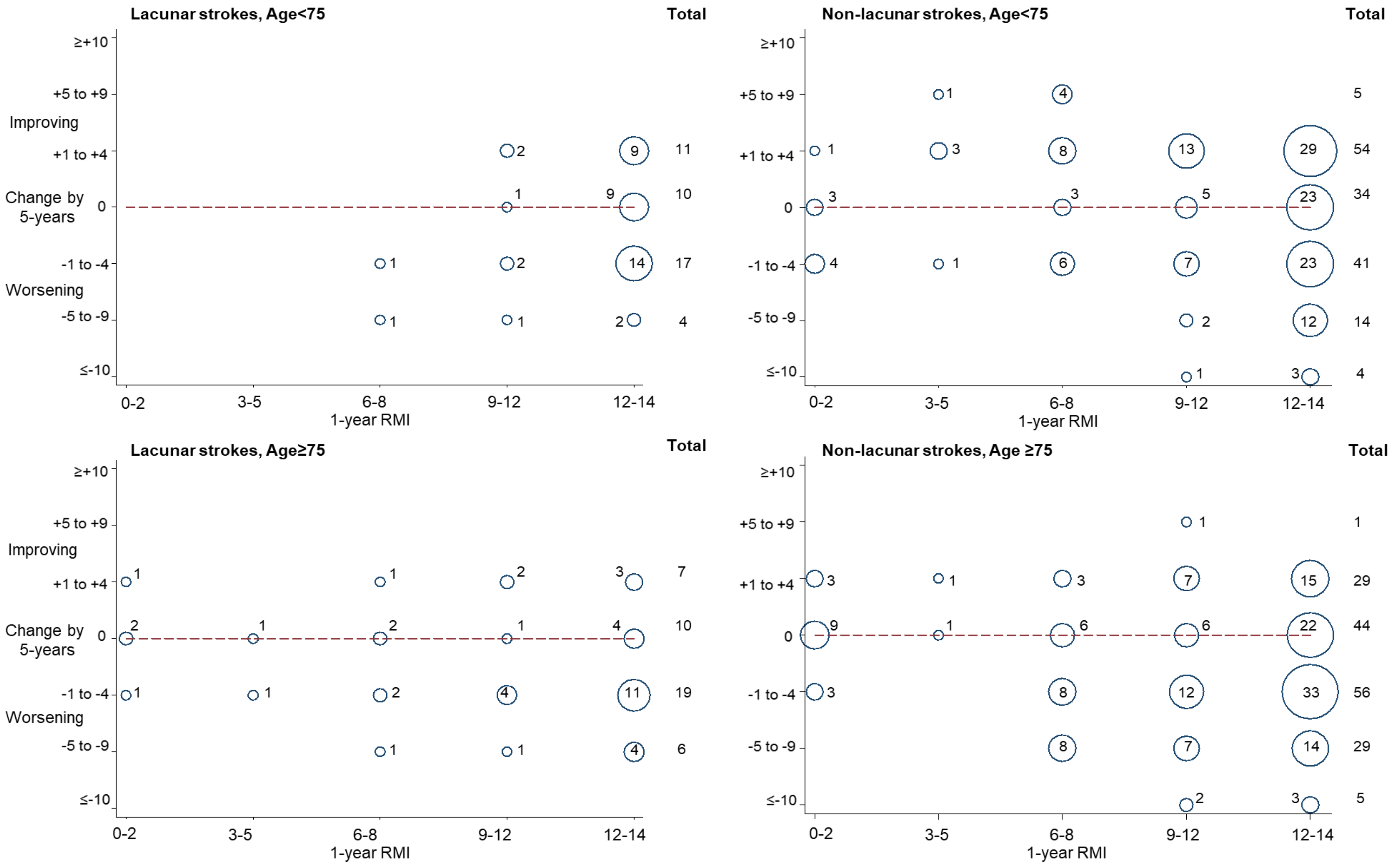

Figure XIII. Changes in RMI between 1-year and 5-years post-stroke for 5-year survivors with lacunar and non-lacunar stroke, stratified by age<75 and ≥75. The size of each bubble represents the number of patients at that intersection. Bubbles falling along the dashed line indicate no change in RMI. Bubbles falling below this line indicate a worsening of RMI score in that time period, while those falling above it indicate improvement.

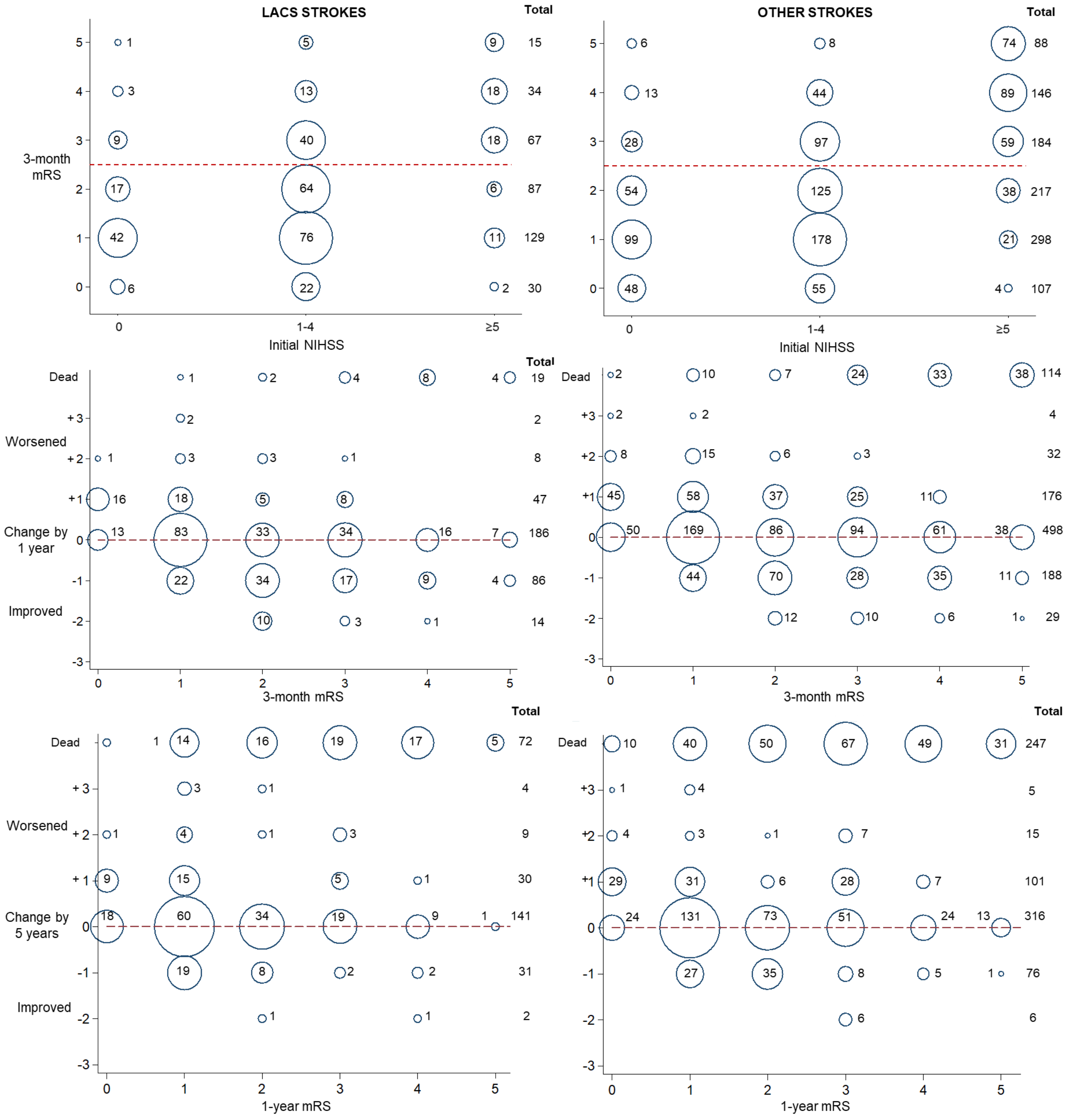

Figure XIV. Early functional improvement (3-month mRS by initial NIHSS) and improvement from 3-months to 1-year, and 1-year to 5-years in 3-month survivors with lacunar stroke syndromes (LACS) versus other strokes. The size of each bubble represents the number of patients at that intersection. Bubbles falling along the dashed line indicate no change in mRS. Bubbles falling above this line indicate a worsening of mRS score in that time period, while those falling below it indicate improvement.
